# Correction: Zhao et al. Isoleucine Enhanced the Function of the Small Intestinal Mucosal Barrier in Weaned Piglets to Alleviate Rotavirus Infection. *Animals* 2024, *14*, 3146

**DOI:** 10.3390/ani16142201

**Published:** 2026-07-15

**Authors:** Rongkun Zhao, Changsheng Jiang, Yuchen Yuan, Shen Zhang, Ahmed H. Ghonaim, Chuanyan Che, Xiaojin Li, Mengmeng Jin, Erhui Jin, Xiangfang Zeng, Shenghe Li, Man Ren

**Affiliations:** 1 Anhui Provincial Key Laboratory of Animal Nutritional Regulation and Health, College of Animal Science, Anhui Science and Technology University, Fengyang 233100, China; rongkunzhao@126.com (R.Z.); jiangcs@ahstu.edu.cn (C.J.); 18855065915@163.com (Y.Y.); 15755797521@163.com (S.Z.); checy@ahstu.edu.cn (C.C.); lixj@ahstu.edu.cn (X.L.); jinmm@ahstu.edu.cn (M.J.); jineh@ahstu.edu.cn (E.J.); 2 National Key Laboratory of Agricultural Microbiology, College of Animal Sciences and Veterinary Medicine, Huazhong Agricultural University, Wuhan 430070, China; a.ghonaim@webmail.hzau.edu.cn; 3 Desert Research Center, Cairo 11435, Egypt; 4 State Key Laboratory of Animal Nutrition, College of Animal Science and Technology, China Agricultural University, Beijing 100193, China; zengxf@cau.edu.cn

In the original publication [1], there was a mistake in Figure 1. Effects of Ile supplementation on small intestinal morphology in RV-infected piglets. Representative histologic HE-stained micrographs. Scale bars are 200 µm. In the original figure, the image representing the RV + 1% Ile group in the duodenum was inadvertently duplicated from the 0.5% Ile group. This mistake has now been corrected by replacing Figure 1 with the accurate version. The corrected Figure 1 appears below. The authors state that the scientific conclusions are unaffected. This correction was approved by the Academic Editor. The original publication has also been updated.

## Figures and Tables

**Figure 1:**
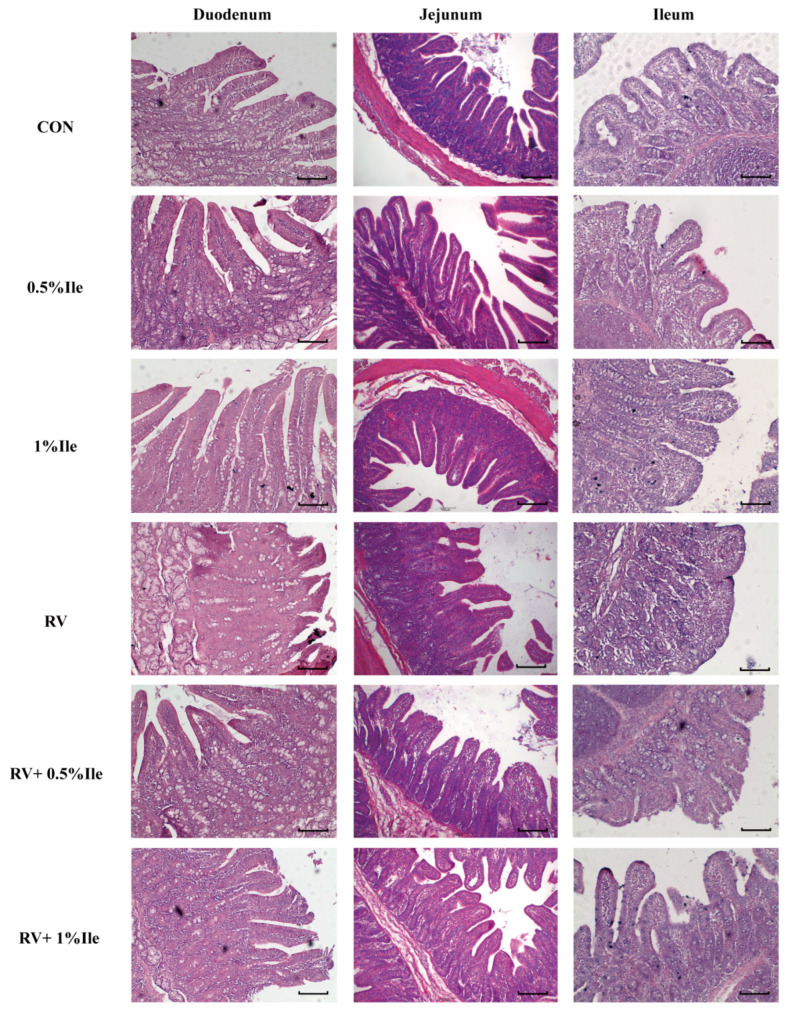
Effects of Ile supplementation on small intestinal morphology in RV-infected piglets. Representative histologic HE-stained micrographs. Scale bars are 200 μm.

## References

[1] Zhao R., Jiang C., Yuan Y., Zhang S., Ghonaim A.H., Che C., Li X., Jin M., Jin E., Zeng X. (2024). Isoleucine Enhanced the Function of the Small Intestinal Mucosal Barrier in Weaned Piglets to Alleviate Rotavirus Infection. Animals.

