# Supplementary material for: Isoleucine Enhanced the Function of the Small Intestinal Mucosal Barrier in Weaned Piglets to Alleviate Rotavirus Infection
Source: Animals (Basel). 2024 Nov 2;14(21):3146. doi: 10.3390/ani14213146 (PMC11545378; doi:10.3390/ani14213146)

β-Actin

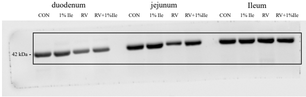

duodenum
jejunum
Ileum
CON
1% Ile
RV
RV+1%Ile
CON
1% Ile
RV
RV+1%Ile
CON
1% Ile
RV
RV+1%Ile
42 kDa

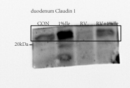
duodenum Claudin 1
CON
1%Ile
RV
RV+1%Ile
20kDa

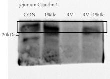
jejunum Claudin 1
CON
1%Ile
RV
RV+1%Ile
20kDa

ileum Claudin 1

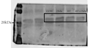

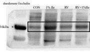
duodenum Occludin
CON
1% Ile
RV
RV+1%Ile
50kDa

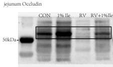
jejunum Occludin
CON
1% Ile
RV
RV+1%Ile
50kDa

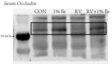

ileum Occludin
CON
1% Ile
RV
RV+1% Ile
50 kDa

duodenum Mucin1

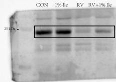

jejunum Mucin 1

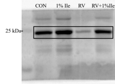

ileum Mucin 1

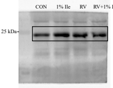

Supplement: Supplementary file 1 [file animals-14-03146-s001.zip › animals-3230260-supplementary.pdf]
